# Metagenomic Analysis Reveals the Effects of Microplastics on Antibiotic Resistance Genes in Sludge Anaerobic Digestion

**DOI:** 10.3390/toxics12120920

**Published:** 2024-12-19

**Authors:** Zhonghong Li, Donghai Yuan

**Affiliations:** School of Environment and Energy Engineering, Beijing University of Civil Engineering and Architecture, Beijing 100044, China; pl_lizhh@163.com

**Keywords:** microplastics, antibiotic resistance genes, metagenome, anaerobic sludge digestion, horizontal gene transfer

## Abstract

Sewage sludge is recognized as both a source and a reservoir for antibiotic resistance genes (ARGs). Within an anaerobic digestion (AD) system, the presence of microplastics (MPs) has been observed to potentially facilitate the proliferation of these ARGs. Understanding the influence of MPs on microbial behavior and horizontal gene transfer (HGT) within the AD system is crucial for effectively managing the dissemination of ARGs in the environment. This study utilized metagenomic approaches to analyze the dynamics of various types of ARGs and potential microbial mechanisms under exposure to MPs during the AD process. The findings indicated that MPs in the AD process can enhance the proliferation of ARGs, with the extent of this enhancement increasing with the dosage of MPs: polyethylene (PE), polyethylene terephthalate (PET), and polylactic acid (PLA) MPs increased the abundance of ARGs in the anaerobic digestion system by up to 29.90%, 18.64%, and 14.15%, respectively. Additionally, the presence of MPs increased the relative abundance of mobile genetic elements (MGEs) during the AD process. Network correlation analysis further revealed that plasmids represent the predominant category of MGEs involved in the HGT of ARGs. *Propionibacterium* and *Alicycliphilus* were identified as the primary potential hosts for these ARGs. The results of gene function annotation indicated that exposure to MPs led to an increased the relative abundance of genes related to the production of reactive oxygen species (ROS), alterations in membrane permeability, ATP synthesis, and the secretion of extracellular polymeric substances (EPS). These genes play crucial roles in influencing the HGT of ARGs.

## 1. Introduction

The excessive utilization of antibiotics has precipitated a surge in the abundance of antibiotic resistance genes (ARGs) and antibiotic-resistant bacteria (ARB) within environmental contexts, posing significant risks to human health and the integrity of global aquatic ecosystems. Statistical data indicate that from 1990 to 2021, antimicrobial resistance (AMR) was responsible for over one million deaths annually worldwide, and the number could rise to nearly two million by 2050. Projections indicate that, within the next 25 years, AMR could be responsible for over 39 million deaths worldwide, attributable to infections caused by resistant microbes [1]. Wastewater treatment plants (WWTPs) serve as critical nodes in the dissemination and retention of ARGs, representing one of the primary human-mediated pathways for introducing ARGs and ARB into the environment [2]. During the wastewater treatment process, the significant translocation of ARGs from wastewater to activated sludge through sedimentation and adsorption leads to their accumulation in sewage sludge (SS) and subsequent discharge into the environment. As a consequential byproduct of the wastewater treatment process, SS is characterized by its high microbial density and nutrient richness, serving as a fertile ground for the proliferation and horizontal gene transfer (HGT) of ARGs [3]. For instance, Wang et al. found that the total content of ARGs in dewatered sludge was 7 to 308 times higher than that in influent and 16 to 638 times higher than that in effluent [4]. Xu et al. identified 202 different types of ARGs in SS using high-throughput quantitative PCR, with their abundance ranging from 3.1 × 10^9^ to 1.2 × 10^10^ copies/g dry weight [5]. Guo et al. conducted a broad-spectrum gene analysis of aerobic activated sludge from a WWTP in Beijing using metagenomic technology, and a total of 42 ARGs subtypes were detected [6]. Consequently, the contamination of SS with ARGs warrants careful attention.

As an emergent class of contaminants, ARGs display environmental behaviors that are distinct from those of traditional pollutants, primarily due to their unique biological properties. ARGs can propagate among microbial communities through mechanisms such as HGT, a process that is significantly influenced by mobile genetic elements (MGEs) [7]. These elements, which facilitate the transfer of various ARGs, include integron (In), insertion sequences (IS), integrating conjugative elements (ICEs), plasmids, and transposons (Tn) [8]. The ability of nearly all functional genes to undergo HGT underscores the heightened environmental health risks associated with ARGs, making their study in environmental settings a critical focal point of concern.

Other emerging contaminants (ECs) such as microplastics (MPs) are frequently detected in SS, posing significant safety risks to the treatment and safe utilization of sludge and threatening human health and ecological environmental quality [9,10,11]. MPs are defined as plastic fragments with a diameter of less than 5 mm and have the potential to leach chemicals that may cause endocrine disruption, carcinogenicity, or mutagenicity in environmental organisms into the environment [12]. WWTPs act as a primary conduit for the entry of MPs into surface water systems, with an estimated 30 to 230 billion microplastic particles (averaging 130 billion) discharged daily into sewers through municipal wastewater and subsequently processed at these WWTPs in Lower Saxony, Germany [13]. Secondary and tertiary treatment processes at WWTPs can effectively remove most MPs from wastewater. However, up to 75.7% of these MPs is subsequently transferred to SS [14]. The surface of MPs can provide a substrate for microbial adhesion and buoyancy, thereby altering the migration and diffusion behavior of ARGs [15]. For instance, Wu et al. discovered that polyvinyl chloride (PVC) MPs form specific microbial biofilm structures on their surfaces, selectively enriching ARGs and bacterial pathogens [16]. Su et al. found that long-term aging significantly alters the surface properties of MPs in leachate, enhancing their potential to enrich ARGs [17]. Consequently, the prolonged presence of MPs in SS amplifies their role as carriers of ARGs, increasing the risk of their propagation and diffusion. Current research predominantly suggests that ARGs in the AD system spread primarily through HGT, yet there is a dearth of studies on the synergistic actions between ARGs and various MGEs and a lack of exploration into the AD process’s capacity to reduce core MGEs.

SS contains a substantial amount of organic matter, such as proteins, polysaccharides, and lipids, which constitute 50% to 70% of its content. AD technology facilitates the biotransformation of organic substances in suspended solids into valuable energy resources, such as methane and hydrogen. Furthermore, the AD process not only effectively controls and reduces the presence of ECs in SS but also exerts a significant lethal effect on the pathogenic microbes contained therein. This establishes AD as the most effective ecological method currently available for sludge treatment [18]. However, MPs adsorbed on the surface of SS can influence microbial activity or degrade under the influence of anaerobic microbes during anaerobic fermentation, thereby impacting critical processes such as hydrolysis, acidogenesis, hydrogen production, and methane generation [19]. For instance, Wei et al. demonstrated that high concentrations of polyethylene (PE) MPs (100–200 particles/g-TS) exposed in the AD of SS resulted in a decrease of up to 15% in hydrolysis rate and 27.5% in methane yield [20]. However, the mechanistic role of MPs in influencing changes in ARGs during the AD of SS remains unclear, underscoring the need for further detailed investigation.

In the realm of research methodologies for ARGs, prior studies have predominantly relied on pure culture techniques, which are somewhat removed from application to complex AD system. Furthermore, contemporary research often employed quantitative polymerase chain reaction (qPCR) techniques to detect specific target ARGs (such as tetracycline and sulfonamide ARGs) within samples. However, the efficacy of this technology is limited by the effectiveness of the amplification primers, allowing only for the quantification of known genotypes of ARGs and precluding the discovery of novel ARGs. Consequently, the results inherently possess a degree of presupposition [21]. Metagenomics technology has increasingly contributed to a comprehensive exploration of the distribution of ARGs in SS. Unlike methods dependent on PCR amplification, metagenomics approaches study the genomic DNA of all microbes present in digested sludge, enabling the rapid, simultaneous acquisition of comprehensive data on all ARGs, related MGEs, and potential host genes within a sample [22]. The results are instrumental for systematically investigating the distribution characteristics and dynamic patterns of ARGs, MGEs, and potential hosts in digested sludge, providing robust support for an in-depth understanding of the broad distribution and study of ARGs in complex environments.

This study employed metagenomic technology to thoroughly investigate the impact of MPs exposure on the distribution tendencies of ARGs, MGEs, and their potential hosts during the AD of SS. It aims to elucidate the fundamental mechanisms that govern the HGT of ARGs, as well as the interactions that affect the environmental fate of ARGs and resistant bacterial strains. These insights are intended to provide essential information for environmental management and policy formulation.

## 2. Material and Methods

### 2.1. Anaerobic Sludge Digestion Experiments

Batch experiments, characterized by their short experimental cycles and straightforward, flexible operation, were employed in this study to investigate the effects of MPs on the variations of ARGs during the AD process of SS. The substrate sludge and inoculum sludge used in this research were both sourced from a WWTP in Beijing. Upon retrieval, the substrate sludge was allowed to naturally settle for 24 h to concentrate and then stored at 4 °C. When needed, tap water was added to adjust the sludge to approximately 7% total solids (TSs) for homogenization.

Polyethylene (PE), polyethylene terephthalate (PET), and polylactic acid (PLA) MPs with an approximate particle size of 100 μm were procured from Chengxin Company. The AD reactions were conducted in 500 mL serum bottles, where the substrate and inoculum sludge were combined in a volumetric ratio of 1:2 and introduced into the bottles. Concentrations of PE, PET, and PLA-MPs were, respectively, set at 5, 50, and 200 mg·L^−1^, with one set of parallel experiments established for each type. The initial pH values of each reactor were adjusted to approximately 7.0 using 1 M HCl and NaOH. High-purity nitrogen gas (>99.99%) was then flushed through each reactor for 2 min to ensure an anaerobic environment, followed by immediate sealing with silicone stoppers and aluminum caps. The anaerobic digesters were placed in an incubated shaker set at mesophilic conditions (37 ± 1 °C) and 150 rpm.

### 2.2. DNA Extraction

Solid samples were subjected to lyophilization before nucleic acid extraction. DNA extraction was performed for each sludge sample using the FastDNA^TM^ Spin Kit for Soil (MP Biomedicals, Irvine, CA, USA). Subsequently, the quality and integrity of the extracted genomic DNA, as well as the yield and purity of DNA post-PCR amplification, were assessed through 1% agarose gel electrophoresis and quantification using the Qubit 2.0 Fluorometer (Thermo Fisher Scientific, Waltham, MA, USA). These evaluations ensured compliance with the requisite standards for library size before sequencing. The genomic DNA samples were preserved on dry ice and immediately dispatched to Microeco Tech Co., Ltd., Shenzhen, China, for sequencing.

### 2.3. Metagenomic Sequencing

Utilizing an ultrasonic disruptor (Covaris, Woburn, MA, USA), DNA from AD samples that met quality control standards was randomly fragmented into approximately 300 bp segments. This was followed by a series of library construction steps for metagenomics, including end-repair, A-tailing, adapter ligation, purification, and PCR amplification. Subsequently, the sediment samples were subjected to paired-end sequencing on the NovaSeq 6000 high-throughput sequencing platform, yielding 6G of sequence data per sample. This process generated paired-end reads, from which raw data were obtained.

### 2.4. Bioinformatic Analysis

#### 2.4.1. Annotation of ARGs

The raw data were subjected to quality control using Trimmomatic (version 0.36), which facilitated the removal of low-quality sequences [23]. Additionally, Bowtie 2 was employed to align the sequences against the human reference genome GRCh38, thereby excluding human host DNA [24]. Following these procedures, FastQC (version 0.11.9) was utilized to evaluate the appropriateness and efficacy of the quality control measures applied to the samples [25].

In this study, the sequencing techniques leveraged the ARGs-OAP v3.2 analysis pipeline, utilizing the SARGfam database based on the Hidden Markov Model (HMM) to identify and assemble sequences and to classify and characterize the types and subtypes of ARGs both qualitatively and quantitatively. The ARGs-OAP online platform serves as a robust tool for the analysis of ARGs, enabling the rapid identification and quantitative analysis of ARGs from metagenomic datasets. The platform incorporates a structured ARG database (SARG) which includes ARG sequences from the CARD, ARDB, and NCBI-NR databases, facilitating the annotation of 32 categories of ARGs and 2842 subtypes [26]. Sequences that meet the DIAMOND criteria (alignment length ≥ 25 aa, similarity ≥ 80%, e value ≤ 1 × 10^−5^) are considered ARG sequences. And the relative abundances of ARGs were standardized by “ARG copy per cell”. [27].

#### 2.4.2. Identification of MGEs

HGT mediated by MGEs represents a significant mechanism for the transmission, propagation, and dissemination of ARGs in the environment, with a richer presence of MGEs typically indicating an enhanced capability for HGT. Consequently, in this study, the abundance of MGEs serves as an indirect indicator of the capacity for HGT within microbial communities. BlastN (version 2.13.0+) was employed to perform comparative annotations against databases including the integrase database (INTEGRAL, http://integrall.bio.ua.pt/, accessed on 15 January 2024) [28], the plasmid database (NCBI RefSeq Database, http://www.ncbi.nlm.nih.gov/refseq, accessed on 16 January 2024), the transposon database (NCBI RefSeq Database, http://www.ncbi.nlm.nih.gov/refseq, accessed on 16 January 2024) [29], and the integrative and conjugative elements database (ICEberg 2.0, https://bioinfo-mml.sjtu.edu.cn/ICEberg2, accessed on 2 February 2024) [30]. The relative abundances of MGEs were standardized by “MGE copy per cell”.

#### 2.4.3. Taxonomic Classification and Functional Annotation

Kraken2 (version 2.0.7-beta) was applied to analyze the microbial community structure of all samples [31]. The sequencing data were assembled using the MEGAHIT (version 1.2.9) [32] tool and then the microbial metabolic functional classes were annotated using the Kyoto encyclopedia of genes and genomes (KEGG) [33].

#### 2.4.4. Statistical Analysis and Visualization

SPSS (version 24.0, SPSS Inc., Chicago, IL, USA) was used for one-way ANOVA analysis of the ARGs, MGEs, and microbial community. Correlation analyses among the ARGs, MGEs, and microbial communities were conducted using the Spearman method, with significance denoted at *r* > 0.7 and *p* < 0.05. Network analysis was performed using Gephi software (version 0.9.2). Origin software (version 2022, OriginLab Corp., Northampton, MA, USA) was constructed to display the predominant types of ARGs and their subtypes in the AD system.

## 3. Results and Discussion

### 3.1. The Effects of MPs on ARG Abundances

**Figure 1:**
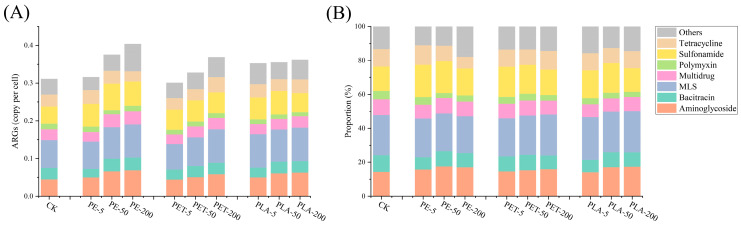
(**A**) Distribution and (**B**) proportion of ARGs in AD system with different dosages and types of MPs.

Metagenomic analysis was conducted to comprehensively explore the impact of PE, PET, and PLA-MPs on the abundance of ARGs throughout the AD process of SS. A total of 26 types of ARGs were detected within the AD system. Among these, macrolide–lincosamide–streptogramin (MLS), aminoglycoside, bacitracin, multidrug, polymyxin, sulfonamide, and tetracycline resistance genes were identified as the predominant ARG types (Figure 1). Exposure to PE, PET, and PLA resulted in significant increases in the relative abundance of ARGs at varying levels compared to the control without MPs (one-way ANOVA, F = 23.257, *p* < 0.05), with a general trend of increased total ARG abundance correlating with higher concentrations of these MPs, particularly at 200 mg/L for PE and PET. In the control group, the total relative abundance of ARGs was 0.311 copies per cell. Under PE exposure, the total relative abundance of ARGs increased to 0.316 copies per cell at 5 mg/L, 0.375 copies per cell at 50 mg/L, and 0.404 copies per cell at 200 mg/L, marking a maximum increase of 29.90%. Under PET exposure, the total abundance of ARGs decreased to 0.301 copies per cell at 5 mg/L but rose to 0.328 copies per cell at 50 mg/L and 0.369 copies per cell at 200 mg/L, achieving a maximum increase of 18.64%. Under PLA exposure, the total relative abundance of ARGs increased to 0.362 copies per cell at 5 mg/L, decreased to 0.355 copies per cell at 50 mg/L, and further to 0.353 copies per cell at 200 mg/L, with a maximum increase of 14.15%. Similar results indicated that Zhang et al. found the relative abundance of eARGs of the eight tests was 1.70 times and 2.15 times that of the control group, respectively, with the exposure of microplastic fibers during sludge anaerobic digestion [34]. Luo et al. found that compared with the control group, the MPs with ARGs abundance in the range of 10~80 particles/g-TS were dose-dependent, and the relative abundance of ARGs increased by 4.5~27.9% [35].

Overall, while individual types of ARGs experienced declines under the exposure to PE, PET, and PLA-MPs, there was a general increase in the total relative abundance of ARGs, with PE exposure resulting in significant higher relative increase compared to PET and PLA (*p* < 0.05). Relative to the control, the addition of these three types of MPs promoted the proliferation of MLS, aminoglycoside, and sulfonamide resistance genes yet exerted an inhibitory effect on polymyxin resistance genes. Metagenomic sequencing offers a comprehensive tool for delineating the spectrum of ARGs during the AD process. MLS resistance genes confer bacterial resistance to MLS antibiotics, a class commonly used to treat a variety of infections including respiratory, skin, and soft tissue infections. Resistance is mediated through multiple mechanisms, including drug efflux, target modification, and enzyme-mediated drug degradation [36]. Aminoglycosides are a broadly utilized class of antibiotics, encompassing drugs such as gentamicin, kanamycin, and amikacin. Aminoglycoside resistance genes enable bacteria to resist these drugs through several mechanisms, including enzyme-mediated drug modification (e.g., phosphorylation, acetylation, or adenylation), drug efflux, and target modification [37]. Sulfonamides are another widely used class of antibiotics, primarily for treating bacterial infections such as urinary and respiratory tract infections. Sulfonamide resistance genes primarily operate by encoding alterations in the antibiotic target dihydropteroate synthase, rendering it insensitive to sulfonamide drugs, or by enhancing the bacterial utilization of environmental folate, thereby circumventing drug inhibition. These genes, such as *sul*1, *sul*2, and *sul*3, are typically located on bacterial plasmids, facilitating their propagation among different bacterial species through HGT [38]. These three types of ARGs are commonly detected in the environment, particularly in hospital wastewater, livestock wastewater, and urban sewage. This prevalence is largely due to the extensive use of aminoglycosides, macrolides, lincosamides, streptogramins, and sulfonamides, incomplete drug metabolism, and insufficient waste treatment systems, which contribute to the widespread distribution and propagation of these ARGs across various environments. The presence and dissemination of these ARGs heighten the proportion of multidrug-resistant bacterial strains in the environment, necessitating enhanced monitoring of these ARG types to assess their spread and ecological impact.

**Figure 2:**
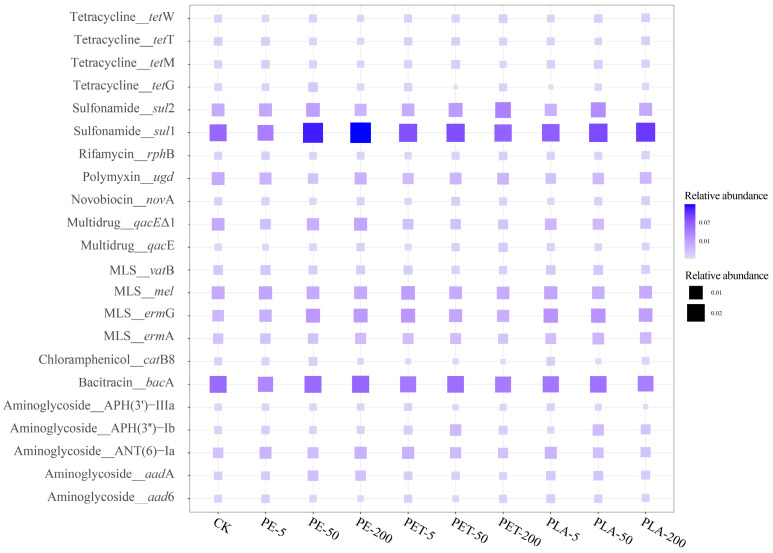
Distribution of ARG subtypes in AD system with different dosages and types of MPs.

In all samples analyzed, 429 subtypes of ARGs were detected, with 22 of these ARGs exhibiting a relative abundance exceeding 0.001 copies per cell (Figure 2). Among the detected ARGs, the sulfonamide resistance gene *sul*1 showed the highest relative abundance, followed by the bacitracin resistance gene *bac*A, the MLS resistance gene *erm*G, another sulfonamide resistance gene *sul*2, and the MLS resistance gene *mel*. The resistance mechanisms associated with these ARG subtypes primarily involve antibiotic inactivation, antibiotic efflux, antibiotic target protection, antibiotic target alteration, and antibiotic target replacement. Notably, *sul*1 demonstrated significant proliferation across various AD systems. Under exposure to PE-MPs, the relative abundance of *sul*1 increased from 0.0182 copies per cell to 0.0277 copies per cell at 50 mg/L PE-MPs and to 0.0297 copies per cell at 200 mg/L PE-MPs. Under PET-MPs exposure, the relative abundance of *sul*1 rose to 0.2414 copies per cell at 5 mg/L PET-MPs, 0.0217 copies per cell at 50 mg/L PET-MPs, and 0.0190 copies per cell at 200 mg/L PET-MPs. Under PLA-MPs exposure, the relative abundance of *sul*1 increased to 0.0196 copies per cell at 5 mg/L PLA-MPs, 0.0222 copies per cell at 50 mg/L PLA-MPs, and 0.0245 copies per cell at 200 mg/L PLA-MPs. The *sul*1 gene is frequently found in integrons, particularly in those associated with multidrug-resistant bacteria in hospitals and communities. Within integrons, the *sul*1 is often located at the end of gene cassettes, which facilitates its stability and propagation [39]. This suggests that MPs exposure under AD conditions enhances the abundance of MGEs, particularly integrons. Furthermore, the presence of *sul*1 not only confers resistance to sulfonamide antibiotics but may also facilitate the co-transmission of other ARGs, thereby increasing bacterial capacity for multidrug resistance. Additionally, due to its high mobility and pathogenicity, *bac*A is classified as a high-risk ARG (Category 1) [40]. Under exposure to PE-MPs, the relative abundance of *bac*A increased by 0.30% at 50 mg/L PE-MPs and by 3.29% at 200 mg/L PE-MPs while showing a decreasing trend under PET and PLA-MPs exposure. Overall, prolonged exposure to MPs, particularly at higher concentrations, promotes the generation and propagation of ARGs, accompanying an elevated risk of ARG proliferation and dissemination.

### 3.2. The Effects of MPs on MGE Abundances

**Figure 3:**
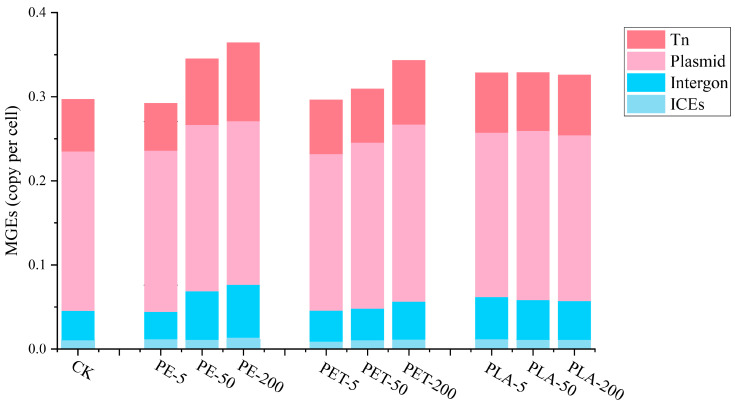
Distribution and of MGEs in AD system with different dosages and types of MPs.

HGT among microorganisms can be facilitated by MGEs such as integrons, transposons, and plasmids, which significantly contribute to the dissemination of ARGs [41]. MGEs serve as crucial indicators for monitoring the lateral transfer of ARGs and are employed in various environments to confirm the capability of ARGs to transfer horizontally [42]. Through alignment with an MGEs database, a total of 263 subtypes of integrons, 87 subtypes of ICEs, 4833 subtypes of plasmids, and 164 subtypes of transposons were annotated (Figure 3). In different AD systems, plasmids exhibited the highest relative abundance among MGEs, followed by transposons, while integrons and ICEs showed lower relative abundances. Furthermore, exposure to various MPs also significantly increased the relative abundance of MGEs within AD systems (one-way ANOVA, F = 43.524, *p* < 0.05). Under exposure to PE and PET-MPs, the relative abundance of MGEs gradually increased with the level of exposure than PLA-MPs, suggesting that PE and PET-MPs may facilitate the increase in MGEs within microbial communities through physical impacts or the release of chemical substances, potentially exacerbating the spread of ARGs. This phenomenon may be related to the chemical properties of the MP surfaces or the microbial adhesion capabilities, as MP surfaces might provide a new ecological niche for microorganisms, thereby influencing their genetic structure. In contrast, exposure to PLA-MPs did not lead to an increase in the relative abundance of MGEs, even with increased PLA concentrations. This may be due to the biodegradable nature of PLA, which does not persist in the environment as long as PE and PET, thus exerting less impact on microbial communities. Additionally, PLA may not facilitate the propagation of MGEs, potentially related to its physical structure or the nature of microbial interactions with PLA [43].

**Figure 4:**
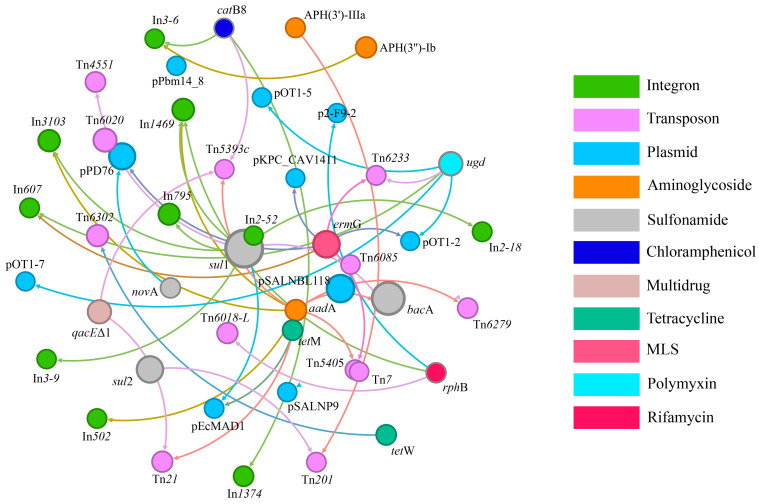
Network analysis of the correlation between ARGs and MGEs.

The dissemination of ARGs in the environment is often mediated by MGEs, which facilitate the HGT of ARGs among microbial populations [44]. The network of correlations between ARGs and MGEs is depicted in Figure 4. In this figure, nodes (circles) represent different subtypes of ARGs or MGEs, while edges (lines) indicate the associations between them. Nodes of different colors signify various subtypes of ARGs or MGEs. Notably, *sul*1 is associated with the integron In*2-18*, the transposon Tn*6279*, and the plasmid p*SALNBL1* (*r* > 0.77, *p* < 0.05), suggesting that the *sul*1 gene may propagate within bacterial populations through the In*2-18* integron. The gene *sul*2 is linked with transposons Tn*21* and Tn*201* (*r* > 0.78, *p* < 0.05), and *erm*G is associated with p*KPC_CAV1411*, Tn*6233*, and In*607* (*r* > 0.77, *p* < 0.05), indicating that ARGs can be disseminated via multiple MGEs. Furthermore, *aad*A and *bac*A are both related to the plasmid p*SALNBL118* (*r* > 0.83, *p* < 0.05), implying that multiple ARGs may coexist within the same host microorganism or on the same MGE, and that HGT may facilitate the spread of ARGs. Integrons such as In*2-18* and In*607* represent a significant class of MGEs capable of integrating and carrying multiple ARGs, forming so-called “gene cassettes”. These gene cassettes can be transferred between bacteria through transposition, significantly enhancing the diffusion of ARGs [45]. Transposons like Tn*6085* and Tn*2010* provide another mechanism by which ARGs can be excised and integrated into different genomes, facilitating their movement from one genome to another [46]. Plasmids such as p*SALNBL1*, p*KPC_CAV1411*, and p*SALNBL118* are circular DNA molecules in bacteria that can replicate autonomously and be transferred between bacteria via conjugation. Plasmids often carry multiple resistance genes, making them crucial vectors for the HGT of ARGs [47].

Research demonstrates that the biofilm environment on MP surfaces enhances the exchange of MGEs such as plasmids and transposons, which frequently carry ARGs [48]. The dense proximity of bacteria within these biofilms significantly increases the opportunities for the exchange of such elements. Within this microenvironment, ARGs can be disseminated among diverse bacterial populations through mechanisms including the insertion of transposons and the conjugative transfer of plasmids. This facilitation of gene transfer underscores the role of MPs as a vector in the propagation of ARGs within microbial communities.

### 3.3. The Effects of MPs on Microbial Communities

**Figure 5:**
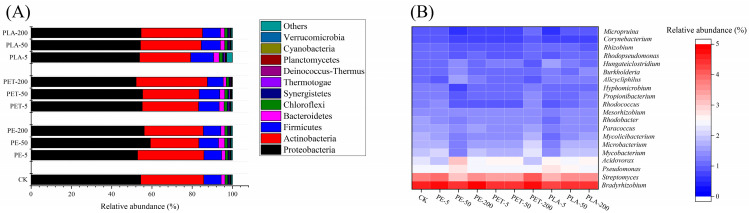
Relative abundance of bacterial community structure at the phylum (**A**) and genus (**B**) level in the AD system.

Research indicates that the presence of MPs may alter the existing microbial community structure, promoting the proliferation of bacteria that are capable of surviving on plastic surfaces and potentially carrying ARGs [49]. To investigate the dynamic response of microbial communities to PE, PET, and PLA-MPs during the AD process, analyses of bacterial and archaeal communities were conducted (Figure 5). The data reveal that the dominant bacterial phyla include Proteobacteria (52.16~59.23%), Actinobacteria (24.06~35.32%), Firmicutes (8.61~11.40%), Bacteroidetes (1.54~3.03%), and Chloroflexi (1.29~1.69%). During the AD process, many species within Proteobacteria decompose complex organic substances by initially breaking down large organic molecules such as proteins, fats, and carbohydrates into smaller molecules like sugars, amino acids, and fatty acids [50]. Actinobacteria primarily participate in the preliminary decomposition of certain types of complex organic matter in anaerobic digestion systems, with some capable of degrading recalcitrant substances such as cellulose and lignin under anaerobic conditions [51]. Throughout the AD process, Firmicutes are among the primary producers of volatile fatty acids (VFAs) such as acetate, propionate, and butyrate. These VFAs are crucial precursors for methane production, playing a vital role in the methanogenic activity of archaea [52].

At the genus level, dominant bacterial genera include *Bradyrhizobium* (4.32~4.83%), *Streptomyces* (3.02~4.11%), *Pseudomonas* (2.35~2.68%), *Acidovorax* (1.87~3.12%), and *Mycobacterium* (1.30~2.23%). *Bradyrhizobium*, typically linked to nitrogen fixation in plant roots, also plays a role in transforming organic nitrogen in anaerobic environments, thereby potentially enhancing the system’s nitrogen cycling efficiency [53]. Streptomyces, a genus of soil bacteria known for decomposing complex organic materials like cellulose and lignin, may facilitate primary decomposition within anaerobic digestion systems, enabling other microorganisms to further utilize the breakdown products [54]. *Pseudomonas* is a genus with a robust metabolic capacity, capable of degrading a wide array of organic pollutants, including some recalcitrant compounds and MPs [55]. For instance, *Pseudomonas sp.* can degrade polyethylene and polypropylene, commonly used in manufacturing plastic bags and containers. Exposure to low concentrations of MPs has been shown to significantly increase the relative abundance of *Pseudomonas* (0.025%~0.17%), whereas high concentrations of MPs inhibit it (−0.14%~−0.10%) (one-way ANOVA, F = 5.72, *p* < 0.05). Low concentrations of MPs may trigger stress responses in *Pseudomonas*, thereby enhancing its metabolic activity and adaptability. Conversely, high concentrations can release chemicals, such as additives or plasticizers, that prove intolerable or toxic to the bacteria, thereby inhibiting their growth [56]. *Acidovorax*, commonly found in aquatic environments and soil, is believed to play a significant role in biodegradation and pollutant treatment. Within anaerobic digestion processes, these bacteria may enhance the stability and efficiency of the system [57].

**Figure 6:**
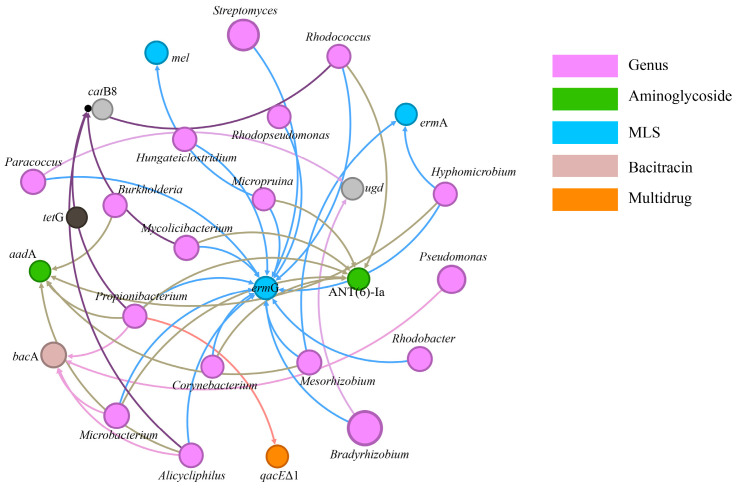
Network analysis of the correlation between ARGs and genus.

To analyze the potential microbial hosts of ARGs and to explore the intrinsic connections between ARGs and bacterial communities, a network analysis of ARGs and bacterial communities was conducted. As depicted in Figure 6, a significant positive correlation exists between 20 bacterial genera and 23 ARGs. This network analysis depicts interactions between various bacterial species and ARGs. In this figure, each node represents either a species or an ARG. The color of each node denotes the genus to which the species or ARG belongs, while the size reflects their relative abundance. These microbial groups are predominantly concentrated in genera such as *Bradyrhizobium*, *Streptomyces*, *Pseudomonas*, *Acidovorax*, *Mycobacterium*, *Microbacterium*, *Mycolicibacterium*, *Paracoccus*, *Rhodobacter*, *Rhodococcus*, *Propionibacterium*, *Hyphomicrobium*, *Alicycliphilus*, *Burkholderia*, *Hungateiclostridium*, *Rhodopseudomonas*, *Rhizobium*, *Corynebacterium*, and *Micropruina*, all of which are core bacteria ubiquitously present in AD reactors. The potential hosts for the *bac*A gene include *Pseudomonas*, *Microbacterium*, *Propionibacterium*, and *Alicycliphilus*. The *erm*G gene has the most potential hosts, showing significant positive correlations with all aforementioned bacterial genera except for *Pseudomonas*, *Acidovorax*, *Mycobacterium*, *Burkholderia*, and *Rhizobium* (*r* > 0.75, *p* < 0.05). Microorganisms known to degrade MPs primarily consist of specific fungi and bacteria, including *Pseudomonas* and *Rhodococcus*. Notably, Rhodococcus, a bacterium recognized for its robust biodegradation capabilities, is capable of decomposing a variety of organic pollutants. This bacterium targets the polymer chains of certain plastics, thereby facilitating their degradation [58]. In the AD system, *Propionibacterium* and *Alicycliphilus* are identified as multidrug-resistant microbes, harboring multiple ARGs. Notably, *Propionibacterium* exhibits significant positive correlations with six ARGs, *bac*A, *qac*EΔ1, *erm*G, *aad*A, and *erm*T, while *Alicycliphilus* shows significant positive correlations with five ARGs: *bac*A, *erm*G, *aad*A, *erm*T, and *erm*F (*r* > 0.72, *p* < 0.05). *Propionibacterium*, an anaerobic or facultatively anaerobic bacterium, is ubiquitously found in various human biomes, including the skin, oral cavities, and intestines [59]. A prolonged and frequent use of antibiotics can lead to the emergence of ARGs within these bacterial communities, including resistance to macrolides, tetracyclines, and beta-lactams. Therefore, during the AD process, exposure to MPs may elevate the risk of transmission of pathogenic bacteria containing ARGs.

### 3.4. Changes in Functional Genes Revealed by Metagenomic Analysis

**Figure 7:**
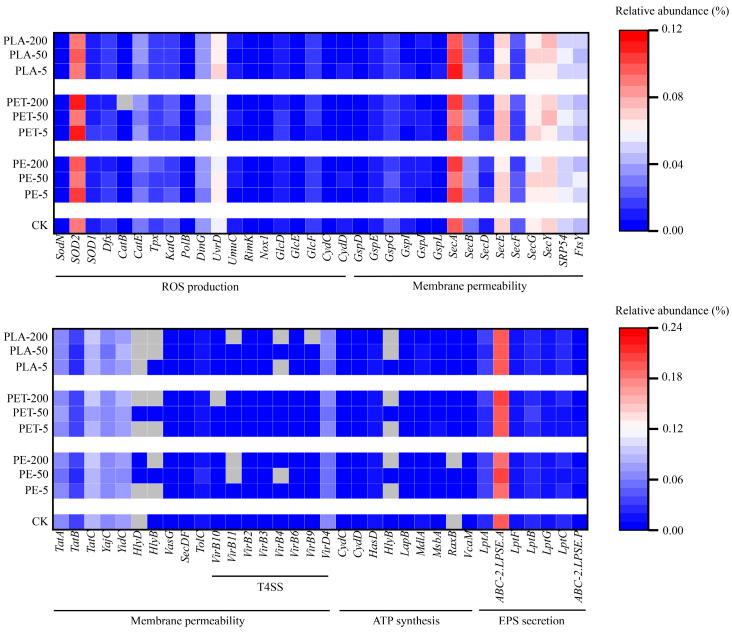
Changes in functional genes in AD system with different dosages and types of MPs.

Based on the aforementioned results, within the AD system, ARGs are disseminated among bacteria via HGT under conditions of MP exposure. Typically, bacterial cells may express or silence specific functionalities contingent upon the survival necessities dictated by varying environmental conditions. The propagation mechanism of ARGs represents a stress response of bacterial cells to antibiotics. For instance, Hu et al. identified potential mechanisms by which MPs facilitate the horizontal mobility of ARGs, including the generation of reactive oxygen species (ROS), alterations in cell membrane permeability, exopolysaccharide (EPS) secretion, and ATP synthesis [60]. Consequently, this study employed metagenomic sequencing to analyze gene functions in samples with different MP amendments and control groups, investigating the functional genes and metabolic pathways correlated with the HGT of ARGs (Figure 7). This approach underscores the intricate interplay between microbial response and environmental stressors, thereby providing a deeper understanding of the mechanisms underpinning ARG dissemination in response to MPs exposure.

ROS, byproducts of cellular metabolism, can be induced by environmental stressors such as exposure to MPs and are capable of damaging DNA, proteins, and lipids, thereby triggering a cellular stress response [61]. For instance, MPs may harbor heavy metals or other oxidative compounds on their surfaces, exacerbating oxidative stress in bacteria. Furthermore, the intrinsic physical structure of MPs may impart mechanical stimuli to bacterial cells, further catalyzing the production of ROS. The SOS response not only facilitates the repair of DNA damage but may also activates MGEs that harbor ARGs [62]. As depicted, exposure to three distinct types of MPs has been shown to enhance the relative abundance of genes related to ROS production. Notably, exposure to 200 mg/L of PE-MPs resulted in the greatest increase in the relative abundance of these genes, a rise of 5.8%. This finding aligns with those of Wei et al., who reported similar effects under comparable conditions [63].

MPs may indirectly facilitate the HGT of ARGs by affecting the permeability of cellular membranes. The permeability of these membranes is a critical determinant in the exchange of cellular materials, including the transfer of MGEs such as plasmids and transposons, which frequently carry ARGs. Initially, the rough surfaces or sharp edges of MPs may physically damage bacterial cell membranes, leading to ruptures or the formation of pores. Additionally, MP surfaces may adsorb harmful chemical substances, including heavy metals and organic pollutants, which can directly compromise the integrity of the cell membrane or alter the fluidity and functionality of membrane lipids [64]. MPs can increase the production of ROS, which themselves can oxidize membrane lipids, leading to lipid peroxidation reactions that disrupt the structure of the cell membrane and increase its permeability. This enhanced membrane permeability may promote the exchange of plasmids and other MGEs carrying ARGs [48]. According to metagenomic data, exposure to PE, PET, and PLA-MPs increased the relative abundance of genes related to membrane permeability in anaerobic digestion systems. Specifically, under exposure of 200 mg/L PE-MPs and 200 mg/L PET-MPs, genes related to membrane permeability increased by 3.53% and 2.66%, respectively, while PLA-MPs showed changes in the relative abundance of genes. The Type IV Secretion System (T4SS) is a ubiquitous secretion system in bacteria that facilitates the transfer of DNA and proteins between bacteria and even between bacteria and host cells. This system plays a pivotal role in the horizontal transfer of ARGs. MPs provide a physical platform that enables bacteria to aggregate and form biofilms on their surfaces. This aggregation increases the frequency of contact between bacteria, potentially enhancing the incidence of T4SS-mediated gene transfer events. The dense colonies formed by bacteria on MP surfaces are particularly conducive to genetic exchanges through systems like T4SS [65,66]. As illustrated in Figure 7, the functional genes related to T4SS are composed of 12 proteins (*VirB1*-*11* and *VirD4*), which facilitate the transport of macromolecules across bacterial cell membranes [67]. Among all T4SS-related functional genes, *VirB3* showed an increase of 182.37% and 97.49% under exposure of 200 mg/L PE-MPs and 200 mg/L PET-MPs, respectively. *VirB3* is a key component of T4SS, involved in constructing and maintaining the structure of this complex protein transport system. Factors affecting the relative abundance of *VirB3* due to MPs exposure could indirectly influence the efficiency of T4SS and the transmission capability of ARGs.

Adenosine triphosphate (ATP) serves as an indispensable energy source for bacterial growth and the maintenance of biofilms. Biofilms on MPs provide a protective environment that enhances the exchange of ARGs among bacteria. Interference from MPs with bacterial energy metabolism, due to altered nutrient availability or toxic substances, may impact ATP production, potentially affecting biofilm stability and gene transfer efficiency [7]. Furthermore, MPs may carry or adsorb a variety of organic and inorganic pollutants, which could act either as substrates or inhibitors in bacterial metabolism. Such interactions could influence the efficiency of ATP synthesis, thereby affecting the overall health of the bacteria and their capacity for gene transfer. As illustrated, exposure to 200 mg/L of PE-MPs and 200 mg/L of PET-MPs resulted in an increase in the relative abundance of genes related to ATP synthesis by 31.72% and 25.48%, respectively. Although ATP synthesis itself is not directly related to the HGT of ARGs, the energetic metabolic state of bacteria can influence their response to environmental stresses, including the stress imposed by MPs and gene transfer activities. This underscores the complex interplay between microbial metabolic processes and environmental factors facilitated by MPs pollution.

EPS are a class of multifunctional high-molecular-weight compounds secreted by bacteria, including polysaccharides, proteins, lipids, and nucleic acids, which play a pivotal role in bacterial biofilm formation and significantly influence adaptability to environmental conditions, ARGs, and HGT [68]. The formation of biofilms is contingent upon the secretion of EPS, which not only provides a structural framework but also aids bacteria in resisting external environmental stresses, including those imposed by MPs. The surface characteristics of MPs, such as roughness and chemical composition, may affect the production and composition of EPS, thereby impacting the integrity and functionality of biofilms. An EPS-rich biofilm environment facilitates close contact between bacteria, creating an ideal setting for HGT through mechanisms such as conjugation, transduction, and transformation. In such microenvironments, ARGs can be more readily propagated within bacterial communities. As illustrated, exposure to 200 mg/L of PE-MPs and 200 mg/L of PET-MPs resulted in a maximal increase in genes related to EPS secretion by 5.16% and 5.32%, respectively. MPs, by altering the secretion and functionality of EPS, indirectly promote or inhibit the transmission of ARGs among bacterial populations. This highlights the intricate interplay between environmental alterations induced by MPs and the genetic dynamics of microbial communities.

## 4. Conclusions

This study employed metagenomic approaches to comprehensively investigate the impact of MP exposure to ARGs, MGEs, and microbial communities within the AD system. The results indicated that exposure to PE, PET, and PLA-MPs enhances the overall abundance of ARGs and MGEs in the AD system, suggesting that the addition of MPs facilitates the proliferation and dissemination of ARGs, with PE-MPs having the most pronounced effect on the spread of ARGs. Furthermore, the succession of bacterial communities and changes in plasmids contribute significantly to the transmission of ARGs. *Propionibacterium* and *Alicycliphilus* are identified as multidrug-resistant bacteria carrying multiple ARGs. MPs influence bacterial behavior and interactions through various mechanisms, thereby affecting the transmission of ARGs. Key functional genes include the generation of ROS, alterations in cell membrane permeability, EPS secretion, and ATP synthesis, which are crucial in the HGT of ARGs influenced by MPs. This research explored the effects of MP exposure on the dynamics of microbial communities, ARGs, and MGEs during the AD process, providing new insights into the mechanisms of ARGs transfer and cross-resistance among microbes.

## Data Availability

The raw data supporting the conclusions of this article will be made available by the authors on request.
